# Working life expectancies among individuals with type 1 and type 2 diabetes over a 30-year period

**DOI:** 10.5271/sjweh.3972

**Published:** 2021-09-30

**Authors:** Mette A Nexø, Jacob Pedersen, Bryan Cleal, Ingelise Andersen, Jakob B Bjørner

**Affiliations:** Steno Diabetes Center Copenhagen, Health Promotion Research, Copenhagen, Denmark; National Research Center for the Working Environment, Epidemiology, Copenhagen, Denmark; Copenhagen University, Institute of Public Health, Section of Social Medicine, Copenhagen, Denmark; QualityMetric Incorporated, Johnston, RI, USA

**Keywords:** cohort study, epidemiology, occupational health, work life expectancy, working years lost

## Abstract

**Objectives:**

This study aimed to (i) estimate working life expectancies (WLE) and the number of working years lost (WYL) among individuals with type 1 and type 2 diabetes over a 30-year period and (ii) identify educational differences in WLE and WYL.

**Methods:**

Individuals aged 18–65 years diagnosed with type 1 (N=33 188) or type 2 diabetes (N=81 930) in 2000–2016 and age- and gender-matched controls without diabetes (N=663 656) were identified in Danish national registers. WLE in years were estimated as time in employment from age 35–65 years. We used a life-table approach with multi-state (eg, disability pension, sickness absence, unemployment) Cox proportional hazard modeling. Analyses were performed separately for sex, cohabitation status, educational duration, and type of diabetes. Inverse probability weights accounted for differences between populations.

**Results:**

People with diabetes had significantly shorter WLE and greater WYL compared to people without diabetes over the 30-year span. At age 35, cohabitant women with lower education and diabetes lost up to 8.0 years [95% confidence interval (CI) 5.0–11.0] and men 7.0 years (95% CI 4.0–8.7). WYL among women with higher education was 4.4 (95% CI 6.6–2.3) and 3.7 years among men (95% CI 1.5–4.5). Compared to people with type 2 diabetes, those with type 1 spend significantly more years in disability pension, but there were no significant differences in the other WYL estimates.

**Conclusions:**

The WYL among people with diabetes is substantial and characterized by social disparities. The WYL help identify intervention targets at different ages, types of diabetes, sex, educational and cohabitant status.

Diabetes mellitus is among the most common non-communicable diseases in the workforce and one of the leading causes of lifelong disability (1). The future workforce will comprise proportionately more individuals with diabetes than is currently the case (2, 3), and the individual and societal implications of this shift require new prevention and management strategies (4).

Work is an important aspect of quality of life and an important setting for daily diabetes management. Diabetes mellitus affects an individual’s ability to work, as indicated by increased risks of short- and long-term sickness absence, unemployment (5–9), and disability pension (10–12). Although adverse work outcomes for people with diabetes are likely to vary across life stages (13), no studies have yet examined diabetes-associated work disability from a life-course perspective.

Similar to life expectancies, working life expectancies (WLE) estimate the number of years populations are expected to work over a defined period of working life, ie, from a specific point in time such as a certain age or when entering the labor force until retirement (14). WLE have been examined in working age populations with mental health issues (15, 16), arthritis (17), poor self-rated health (18), and high physical work demands (19) but has not yet been examined among those with type 1 (T1) or type 2 (T2) diabetes.

Although diabetes-associated work disability is partially explained by disease severity (20), an individual’s health and ability to work are shaped by multiple factors throughout the lifespan (21) and cannot be reduced to the way a disease impacts bodily functions (22). Ideally, studies examining work disability in diabetes should consider how individual (eg, age, gender, education, cohabitation status), and societal factors (eg, access to healthcare, employment opportunities) influence the ability to work. However, most studies examining work disability in diabetes apply cross-sectional designs with limited theoretical frameworks, small sample sizes or prospective designs with limited follow-up periods. In the few studies that include T1 diabetes, it is difficult to identify possible differences between diseases and how these risks may change over time (7–9, 23). One study showed that the elevated risk of sickness absence among women with diabetes remained stable, but the risk for men increased over time (hazard ratio: 1.57–1.82) (9). A recent study applying a multi-state design showed no difference between type 1 and 2 diabetes on a wide range of labor market outcomes but a higher risk of unemployment, sickness absence, and disability pension among men than among women (12).

Many countries, including high-income countries, are facing substantial and growing social disparities in diabetes prevalence. Social gradients, as measured by educational or occupational status, influence both the incidence of T2 diabetes and the extent of morbidity that people with T2 diabetes are likely to experience over time (11, 24–26). People with lower education have a markedly higher risk of T2 diabetes compared to those with higher education (27, 28). These differences are more pronounced among men than women (29). Evidence regarding the incidence of T1 diabetes is conflicting (30), but socioeconomic status also seems to influence morbidity and mortality of T1 diabetes over time (31).

Although cohabitant status may protect people with diabetes from adverse labor market outcomes, it has not yet been investigated. Whereas cohabitant people receive more social support, people living alone are more prone to social isolation, which can have adverse health effects (32). Living alone is associated with higher incidence of T2 diabetes among men and poorer self-management and higher risk of mortality among people with diabetes (33).

Recently, ways of estimating WLE have been tailored to the Danish labor market system, enabling examination of the impact of individual and societal factors on work lifespans at the level of populations (18, 34). Examining WLE in diabetes could both help identify diabetes-associated disability in populations with T1 and T2 diabetes and illuminate occupational health in diabetes from a life-course perspective. Understanding the entire work lifespan of individuals with diabetes can help identify long-term occupational health strategies in diabetes.

The aims of the study were to identify (i): WLE of people with T1 and T2 diabetes, as estimated by the number of years in paid employment from age 35–65 years (the retirement age in Denmark), (ii) the number of working years lost (WYL), as estimated by differences between the number of years in employment of people with T1 and T2 diabetes and those without diabetes, and (iii) social gradients, as measured by educational status, in WLE and WYL among people with T1 and T2 diabetes.

## Methods

In a historical cohort study, we linked several Danish national registers to identify all individuals with T1 and T2 diabetes aged 35–65 years in the Danish population. People with T1 or T2 diabetes were matched to control participants without diabetes in the follow-up period from 1 January 2000 to 31 December 2016.

### Populations

We identified 3 337 314 people aged 18–64 years from the Danish population during the follow-up period. Individuals with T1 diabetes (N=33 188) and T2 diabetes (N=81 930) were age- and gender-matched to control participants without diabetes randomly selected from the general population in a 1:5 ratio (N=663 656).

We identified people diagnosed with diabetes using diagnostic codes from the International Classification of Diseases, version 10 (ICD-10) (35) in the Danish National Patient Register (36) and Anatomical Therapeutic Chemical (ATC) codes in the Danish National Prescription Register (37). These registers include all Danish citizens who have visited any hospital in Denmark and all prescriptions redeemed at any pharmacy in Denmark.

Given that treatment at hospital-based diabetes clinics is standard in the care of T1 diabetes in Denmark, people with T1 diabetes were likely to be identified via the patient register. However, we applied a conservative approach; people were only identified with T1 diabetes if they were registered by ICD-10 diagnosis code E10 and had ≥3 redeemed prescriptions with the ATC code for insulin and analogues (A10A) at any time during the follow-up period. Treatment at the general practitioner is the standard care of people with T2 diabetes (unless they have complications or other complicated comorbidities) and they are, therefore, not necessarily registered with the T2 diagnosis in the Danish National Patient Register. Therefore, we identified people with T2 diabetes either by ICD-10 diagnosis code E11 or by ≥3 redeemed prescriptions with the ATC code for blood glucose-lowering drugs, excluding insulin (A10B). Control participants were defined as having no diabetes if they had no recorded ICD-10 codes (E10-14, diabetes mellitus; O24.4, gestational diabetes mellitus) or ATC codes (A10B or A10A). Participants were included in the study on the date of their earliest recorded diagnosis. Somatic and mental chronic comorbidities previously identified as common in occupational populations were also identified from national patient and prescription registers by diagnostic and ATC codes tested in a previous study (9) (cancer; chronic pain; endocrine, hypertension, heart disease, inflammatory bowel disease, kidney disease, liver diseases, neurological, osteoarthritis; paraplegia and hemiplegia, pulmonary, retinopathy, stroke, dementia, substance abuse, and depression and anxiety).

### Working life expectancies and working years lost

WLE were defined as the expected time in years a person would remain in work from age 35–65 years (the retirement age in Denmark at the time of the study). WYL were defined as the number of years a person was expected to lose during this period.

Each participant’s employment status was identified by the Danish Register-based Evaluation of Marginalization (DREAM) (38). The Danish labor market system is a flexicurity system, characterized by high transition rates between employment and unemployment and easy access to national welfare benefits for Danish residents during temporary (eg, sickness absence, unemployment, maternity leave, education) and permanent periods (disability pension) in which they are unable to work. High levels of income tax finances the welfare system and secures social transfer benefits for all Danish citizen. The right to receive social transfer benefits is secured by law (39).

DREAM covers all residents in Denmark who have received social transfer payments since 1991 and the risk of misclassification is low (40, 41). Payments are recorded on an individual and weekly basis and therefore the DREAM register is well suited for study designs relying on continuous follow-up data. Participants’ employment periods were defined as those during which they received no social transfer payments. Their periods without employment were defined by receipt of social transfer payments for unemployment, long-term sickness absence (payments for a minimum of four consecutive weeks), temporary absence from labor market (payments for maternity leave, education, or emigration), disability pension, or death before age 65.

**Figure 1:**
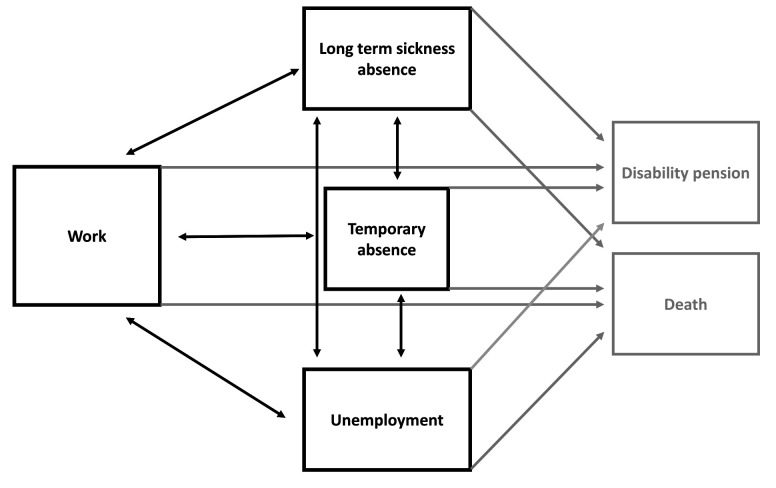
Overview of working life estimation: 12 recurrent transitions between work, sick, unemployment, or temporary absence (transient states in black, 8 permanent states in grey).

From DREAM, we were able to model 12 recurring transitions between transient states of work, illness, unemployment, or temporary absence and on 8 permanent transitions (‘absorbing states’) from work, illness, unemployment, or temporary absence to either disability pension or death (figure 1).

In order to assess the risk of misclassification of participants, who could be out of work but were not registered with a social transfer benefit in DREAM, we linked the DREAM register for 2016 with the yearly disposable income (‘DISPON’) register for 2016 from Statistics Denmark. We used a yearly income of 50 000 DKK as cut-off point of ‘not working’.

Additional information on cohabitation and educational status were obtained from national registers at Statistics Denmark. Participants were categorized as cohabitants if partners were registered at the same address or living alone if no partners were registered. Educational status was defined as the longest educational period completed as registered at age 35 and categorized according to the International Standard Classification of Education 2011 (42): (i) long (bachelor’s degree or equivalent, master’s degree or equivalent, and doctoral degree or equivalent; ≥14 years); (ii) medium (upper secondary education, post-secondary non-tertiary education and short-cycle tertiary education; 11–14 years); (iii) short (early childhood education, primary or lower secondary education; 0–10 years).

All Danish citizens are assigned a unique personal identification number, administered by the Central Population Register (CPR). We used encrypted CPR, accessed via Statistics Denmark, to link data from national registers of health and labor market statistics. The Danish Protection Agency registered and approved the study, identification number: 2014-54-0804.

### Statistical methods

WLE were estimated using state-of-the-art methodology (18) combining a life-table approach, a multi-state design, and the Cox proportional hazard regression model with direct estimation of 95% confidence intervals (CI). WLE estimates were based on 12 recurring transitions between transient states of work, illness, unemployment, or temporary absence and 8 permanent transitions (‘absorbing states’) from work, illness, unemployment, or temporary absence to either disability pension or death (figure 1). Participants who retired early from causes other than disability pension, such as voluntary early retirement, were censored, as were participants who reached age 65 and those at the end of the follow-up period.

Analyses were performed separately for sex, cohabitation status, educational status, and type of diabetes. We examined between-group differences for people with T1 and T2 diabetes and no diabetes (95% CI). In addition to WLE, we estimated expected years of long-term sickness absence, unemployment, temporary absence from the labor market, disability pension, and death. WYL were estimated as the difference between the expected number of years in the listed states of work (figure 1) for people with diabetes and the control participants without diabetes.

Age was the underlying time variable. The time periods of the participants’ different states (eg, work, sickness absence, etc.) were estimated by age, a start-age and an end-age, within a 5-year follow-up period. When summing up all individuals at the ages from 35–65 years, individuals will be registered in the different states (eg, work) within any given small age-interval of 1/365 of a year. The calculation gives the instant transition probability for the particular transition within each of the 5-year age-intervals. Then all the instant transition probabilities for all the possible transitions can be arranged into an instant-transition-matrix within the particular age-interval. By multiplying all the matrixes for each age-interval together, one gets the transition probability for each transition and, in addition, the probability of staying in a particular state. These estimates can all be plotted with age on the x-axis and the transition probability on the y-axis. The area under the curves gives an estimate of the expected time in each state until the age of 65 years, dependent on the starting age (18).

We used inverse probability weights to control for differences between populations in immigrant status and confounding conditions. Retinopathy, hypertension, heart disease and kidney disease (43) inflammatory bowel disease (44), and depression and anxiety (45) are, in relative terms, highly prevalent in diabetes populations. We therefore considered these conditions as complications to, or a consequence of, living with diabetes and, therefore, not confounders. They were not included as inverse probability weights. All analyses were performed with SAS V.9.4 (SAS Institute, Cary, NC, USA).

## Results

Population characteristics are shown in Table 1. Most subjects were of Danish origin and living with a partner. Approximately two thirds of individuals with diabetes and half of those in the control group had one or more comorbidities (table 1).

**Table 1:** Population characteristics.

	Women			Men		
	
	Type 1 diabetes N=12 055	Type 2 diabetes N=34 086	Control group N=277 150	Type 1 diabetes N=21 133	Type 2 diabetes N=47 844	Control group N=386 506
					
	N (%)	N (%)	N (%)	N (%)	N (%)	N (%)
Age intervals (years)						
35–39	4461 (37)	9786 (29)	77 115 (28)	6411 (30)	3367 (7)	54 197 (14)
40–44	1494 (12)	3267 (10)	28 277 (10)	2662 (13)	4419 (9)	39 292 (10)
45–49	1676 (14)	4229 (12)	35 815 (13)	2909 (14)	7264 (15)	56 765 (15)
50–54	1847 (15)	5719 (17)	48 052 (17)	3492 (17)	10 555 (22)	78 064 (20)
55–59	1809 (15)	7297 (21)	58 013 (21)	3768 (1)	13 342 (28)	94 889 (25)
60–64	768 (6)	3788 (11)	29 878 (11)	1891 (89)	8897 (19)	63 299 (16)
Education years a						
Short (≤10)	3738 (31)	9453 (28)	60 310 (22)	5930 (28)	13 963 (29)	83 740 (22)
Medium (11–14)	4991 (41)	14 223 (42)	111 874 (40)	10 224 (48)	23 508 (49)	184 167 (48)
Long (≥14)	3085 (26)	9 778 (29)	101 197 (37)	4569 (22)	9242 (19)	110 968 (29)
Missing	241 (2)	632 (2)	3769 (1)	410 (2)	1131 (2)	7631 (2)
Cohabitation status						
Living with a partner	8880 (74)	25 369 (74)	209 327 (76)	14 607 (69)	34 081 (71)	296 256 (77)
Living alone	3175 (26)	8717 (26)	67 823 (25)	6526 (31)	13 763 (29)	90 250 (23)
Immigration status						
Danish	11 224 (93)	30 304 (89)	262 387 (95)	19 915 (94)	42 809 (90)	365 444 (95)
Descendants	34 (0)	207 (0)	1152 (0)	59 (0)	159 (0)	969 (0)
Immigrant	797 (7)	3575 (11)	13 611 (4)	1159 (6)	4876 (10)	20 093 (5)
Chronic comorbidities b						
Yes	7770 (65)	24 194 (71)	147 590 (53)	12 342 (58)	30 897 (64.6)	183 131 (47)
None	4285 (36)	9892 (29)	129 560 (47)	8791 (42)	16 947 (35.4)	203 375 (53)

a Short: early childhood education, primary education, or shorter secondary education; Medium: upper secondary education, post-secondary non-tertiary education, or short-cycle tertiary education; Long: bachelor’s degree or equivalent, master’s degree or equivalent or doctoral degree or equivalent.

b Chronic comorbidities include cancer; endocrine, neurological, pulmonary, and liver diseases; paraplegia and hemiplegia; osteoarthritis; chronic pain; stroke; dementia; substance abuse

### Working life expectancies

**Figure 2:**
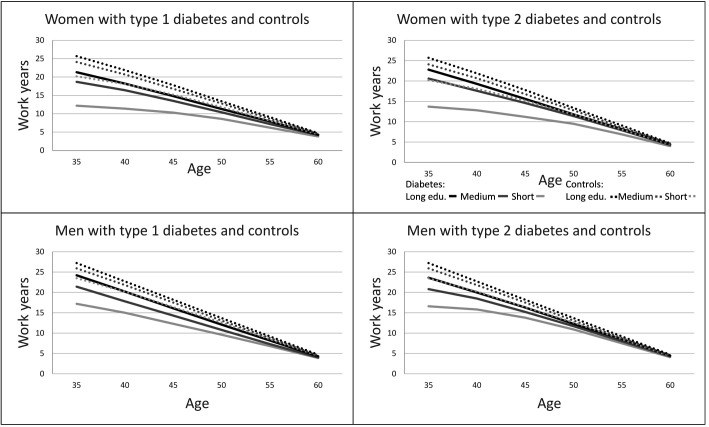
Working life expectancies (WLE) in cohabitant men and women with diabetes and controls by three educational durations (long, medium, short)

Figure 2 is an overview of WLE of cohabitant men and women with T1 and T2 diabetes and the controls, and table 2a shows the estimates of WLE. Supplementary figure S1 (www.sjweh.fi/article/3972) and table 2b give an overview of the same estimates for participants living alone. The figures highlight the educational differences in WLE.

**Table 2 a:** Working life expectancies (WLE) in years and 95% confidence intervals (CI) of **cohabitant men and women** with type 1 and type 2 diabetes and controls by age, and education.

Age	Education a	Women			Men		
	
		Type 1 diabetes	Type 2 diabetes	Controls	Type 1 diabetes	Type 2 diabetes	Controls
					
		WLE (95% CI)	WLE (95% CI)	WLE (95% CI)	WLE (95% CI)	WLE (95% CI)	WLE (95% CI)
35	Short	12.2 (10.7–13.6)	13.7 (12.6–14.9)	20.2 (19.8–20.6)	17.2 (16.0–18.3)	16.6 (15.3–17.8)	23.5 (23.2–23.9)
	Medium	18.7 (17.8–19.7)	20.6 (19.8–21.3)	24.1 (23.9–24.3)	21.4 (20.7–22.0)	20.8 (20.1–21.6)	25.9 (25.8–26.1)
	Long	21.3 (20.2–22.4)	22.8 (22.0–23.6)	25.7 (25.6–25.9)	24.2 (23.5–25.0)	23.6 (22.7–24.4)	27.2 (27.1–27.4)
40	Short	11.4 (10.2–12.6)	12.8 (11.9–13.7)	18.1 (17.8–18.4)	15.0 (14.1–15.9)	15.8 (15.1–16.5)	20.2 (20.0–20.4)
	Medium	16.4 (15.6–17.2)	17.6 (17.0–18.2)	20.7 (20.5–20.8)	17.8 (17.2–18.3)	18.5 (18.0–19.0)	21.8 (21.7–21.9)
	Long	18.2 (17.3–19.1)	19.3 (18.6–19.9)	21.9 (21.8–22.1)	20.2 (19.6–20.8)	20.0 (19.4–20.7)	22.7 (22.6–22.9)
45	Short	10.3 (9.4–11.2)	11.2 (10.6–11.9)	15.2 (15.0–15.4)	12.3 (11.6–12.9)	13.8 (13.3–14.3)	16.4 (16.3–16.6)
	Medium	13.5 (12.9–14.1)	14.6 (14.2–15.1)	16.8 (16.7–16.9)	14.3 (13.9–14.7)	15.2 (14.9–15.6)	17.5 (17.4–17.5)
	Long	14.8 (14.0–15.5)	15.6 (15.0–16.1)	17.8 (17.7–17.9)	16.1 (15.6–16.6)	16.3 (15.8–16.7)	18.2 (18.1–18.3)
50	Short	8.6 (7.9–9.3)	9.5 (9.0–9.9)	11.8 (11.7–12.0)	9.6 (9.1–10.1)	10.9 (10.6–11.3)	12.6 (12.5–12.7)
	Medium	10.4 (9.9–10.9)	11.4 (11.0–11.7)	12.8 (12.7–12.9)	10.8 (10.4–11.1)	11.7 (11.5–11.9)	13.1 (13.1–13.2)
	Long	11.3 (10.7–11.9)	11.8 (11.4–12.3)	13.4 (13.4–13.5)	12.1 (11.7–12.5)	0.7 (0.5–0.9)	13.7 (13.6–13.8)
55	Short	6.2 (5.7–6.7)	6.9 (6.6–7.2)	8.1 (8.1–8.2)	6.8 (6.4–7.2)	7.5 (7.3–7.7)	8.5 (8.4–8.6)
	Medium	7.2 (6.8–7.6)	7.9 (7.7–8.1)	8.6 (8.6–8.7)	7.3 (7.1–7.6)	8.0 (7.9–8.1)	8.8 (8.7–8.8)
	Long	7.9 (7.5–8.3)	8.2 (8.0–8.5)	9.1 (9.0–9.1)	8.2 (7.9–8.5)	8.5 (8.3–8.7)	9.2 (9.1–9.2)
60	Short	3.8 (3.4–4.1)	4.0 (3.8–4.1)	4.4 (4.3–4.4)	3.9 (3.7–4.1)	4.1 (4.0–4.2)	4.4 (4.4–4.5)
	Medium	4.2 (3.9–4.4)	4.3 (4.2–4.4)	4.6 (4.5–4.6)	4.0 (3.9–4.2)	4.3 (4.2–4.3)	4.5 (4.5–4.5)
	Long	4.3 (4.1–4.6)	4.4 (4.3–4.6)	4.7 (4.6–4.7)	4.3 (4.2–4.5)	4.5 (4.4–4.6)	4.7 (4.6–4.7)

a Educational duration: Short: early childhood education, primary education, or Shorter secondary education; Medium: upper secondary education, post-secondary, non-tertiary education, or short-cycle tertiary education; Long: bachelor’s degree or equivalent, master’s degree or equivalent or doctoral degree or equivalent.

**Table 2 b:** Working life expectancies (WLE) in years and 95% confidence intervals (CI) of **men and women living alone** with type 1 and type 2 diabetes and controls, by age and education.

Age	Education a	Women			Men		
	
		Type 1 diabetes	Type 2 diabetes	Controls	Type 1 diabetes	Type 2 diabetes	Controls
					
		WLE (95% CI)	WLE (95% CI)	WLE (95% CI)	WLE (95% CI)	WLE (95% CI)	WLE (95% CI)
35	Short	9.6 (8.2–11.0)	9.6 (8.5–10.7)	15.9 (15.4–16.4)	10.2 (9.1–11.3)	9.0 (7.8–10.1)	15.5 (15.0–16.0)
	Medium	15.0 (14.0–16.1)	17.5 (16.7–18.4)	21.6 (21.4–21.9)	14.8 (14.1–15.5)	14.1 (13.3–15.0)	20.9 (20.7–21.1)
	Long	18.6 (17.4–19.9)	21.3 (20.4–22.2)	23.8 (23.5–24.0)	17.2 (16.0–18.3)	16.6 (15.3–17.8)	23.4 (23.1–23.6)
40	Short	9.1 (8.0–10.3)	9.2 (8.3–10.2)	14.8 (14.4–15.2)	9.4 (8.5–10.3)	9.8 (9.0–10.6)	14.2 (13.9–14.5)
	Medium	13.4 (12.5–14.3)	15.2 (14.5–15.9)	18.8 (18.6–19.0)	12.3 (11.7–12.9)	13.6 (13.0–14.2)	17.8 (17.6–18.0)
	Long	16.0 (15.0–17.0)	17.9 (17.1–18.7)	20.4 (20.2–20.6)	15.0 (14.1–15.9)	15.8 (15.1–16.5)	19.7 (19.5–19.9)
45	Short	8.5 (7.5–9.4)	8.4 (7.7–9.2)	12.9 (12.6–13.1)	7.9 (7.2–8.5)	9.5 (8.9–10.0)	12.0 (11.8–12.3)
	Medium	11.1 (10.4–11.9)	12.7 (12.1–13.2)	15.4 (15.2–15.5)	10.0 (9.5–10.5)	11.6 (11.2–12.0)	14.4 (14.3–14.5)
	Long	13 (12.2–13.8)	14.4 (13.8–15.1)	16.6 (16.4–16.7)	12.3 (11.6–12.9)	13.8 (13.3–14.3)	15.9 (15.8–16.1)
50	Short	7.2 (6.4–7.9)	7.4 (6.9–7.9)	10.2 (10.1–10.4)	6.4 (5.9–7.0)	8.0 (7.6–8.4)	9.6 (9.5–9.8)
	Medium	8.7 (8.1–9.3)	10.0 (9.6–10.4)	11.7 (11.6–11.9)	7.7 (7.3–8.0)	9.2 (8.9–9.5)	10.9 (10.8–11.0)
	Long	10.0 (9.4–10.7)	11.0 (10.5–11.5)	12.6 (12.5–12.7)	9.6 (9.1–10.1)	10.9 (10.6–11.3)	12.0 (11.9–12.2)
55	Short	5.3 (4.7–5.8)	5.6 (5.2–5.9)	7.2 (7.1–7.3)	4.9 (4.5–5.3)	5.8 (5.5–6.0)	6.8 (6.7–6.9)
	Medium	6.1 (5.7–6.6)	7.0 (6.7–7.3)	8.0 (7.9–8.1)	5.4 (5.1–5.6)	6.5 (6.3–6.7)	7.4 (7.3–7.4)
	Long	7.1 (6.7–7.6)	7.7 (7.4–8.0)	8.5 (8.5–8.6)	6.8 (6.4–7.2)	7.5 (7.3–7.7)	8.1 (8.1–8.2)
60	Short	3.3 (3.0–3.7)	3.4 (3.2–3.6)	4.0 (4.0–4.1)	3.2 (2.9–3.4)	3.4 (3.3–3.6)	3.8 (3.8–3.9)
	Medium	3.8 (3.5–4.1)	3.9 (3.8–4.1)	4.3 (4.3–4.4)	3.2 (3.0–3.4)	3.7 (3.6–3.8)	4.0 (3.9–4.0)
	Long	4.1 (3.7–4.4)	4.2 (4.1–4.4)	4.5 (4.4–4.5)	3.9 (3.7–4.1)	4.1 (4.0–4.2)	4.3 (4.3–4.3)

a Educational duration: Short: early childhood education, primary education, or shorter secondary education; Medium: upper secondary education, post-secondary, non-tertiary education, or short-cycle tertiary education; Long: bachelor’s degree or equivalent, master’s degree or equivalent or doctoral degree or equivalent.

The gaps in WLE between long and short periods of education were larger among people with diabetes than controls over the 30-year period. For example, WLE for cohabitant women with T1 diabetes, at age 35, with higher education was 21.3 years and 12.2 for lower education (9.1 years difference), whereas the difference for controls were 5.5 years (long 25.7 and short 20.3 education years, table 2a.)

The shortest WLE were observed among individuals living alone (table 2b). For individuals with T1 diabetes, WLE at age 35 ranged from 12.2 (short) to 21.3 (long) education years for women and from 17.2 (short) to 24.2 years (long) education years for men. For individuals with T2 diabetes, WLE at age 35 ranged from 9.6 (short) to 21.3 (long) education years for women and from 9.0 (short) to 17.2 (long) education years for men. Among people living with a partner, women had lower WLE than men (table 2). The lowest expectancies were observed in those with short education years among people living alone.

### Working years lost

Individuals with both types of diabetes lost significantly more work years compared with matched controls without diabetes throughout the entire work lifespan (table 3a and 3b). Overall, cohabitating people with short education lost the most work years. Women with T1 diabetes with short education years had work lifespans eight years shorter than controls [8.0 (95% CI 11.0–5.0)], decreasing to a loss of four years with long education years [4.4 (95% CI 6.6–2.3)]. Men with T1 diabetes and short education years lost 6 years of work [6.4 (95% CI 8.7–4.0)], but the loss was only 3 years if they had long education years [3.0 (95% CI 4.5–1.5)]. These educational differences were also observed among women but not men living alone. The educational differences were most pronounced among cohabitant men and women and less pronounced among men living alone. We found no significant differences in WYL between T1 and T2 diabetes.

**Table 3a:** Working years lost (WYL) in years and 95% confidence intervals (CI) of **cohabitant men and women** with type 1 (T1) and type 2 (T2) diabetes compared to those without by age and education.

Age	Education a	Women		Men	
	
		T1 compared to controls	T2 compared to controls	T1 compared to controls	T2 compared to controls
			
		WYL (95% CI)	WYL (95% CI)	WYL (95% CI)	WYL (95% CI)
35	Short	8.0 (5.0–11.0)	6.5 (4.0–8.9)	6.4 (4.0–8.7)	7.0 (4.5–9.4)
	Medium	5.4 (3.4–7.3)	3.5 (2.0–5.0)	4.6 (3.3–5.8)	5.1 (3.6–6.6)
	Long	4.4 (2.3–6.6)	2.9 (1.3–4.5)	3.0 (1.5–4.5)	3.7 (2.0–5.4)
40	Short	6.7 (4.3–9.1)	5.3 (3.4–7.2)	5.2 (3.4–7.0)	4.4 (2.9–5.9)
	Medium	4.3 (2.7–5.9)	3.1 (1.8–4.3)	4.0 (2.9–5.1)	3.3 (2.3–4.2)
	Long	3.7 (2.0–5.5)	2.7 (1.3–4.0)	2.5 (1.3–3.7)	2.7 (1.5–4.0)
45	Short	4.9 (3.1–6.7)	4.0 (2.6–5.4)	4.2 (2.8–5.5)	2.6 (1.7–3.6)
	Medium	3.3 (2.0–4.6)	2.2 (1.3–3.1)	3.2 (2.3–4.0)	2.2 (1.6–2.9)
	Long	3.0 (1.5–4.5)	2.2 (1.1–3.3)	2.1 (1.1–3.2)	2.0 (1.1–2.9)
50	Short	3.2 (1.9–4.6)	2.4 (1.4–3.3)	3.0 (2.0–4.0)	1.6 (1.0–2.3)
	Medium	2.4 (1.4–3.4)	1.4 (0.7–2.1)	2.4 (1.7–3.0)	1.4 (1.0–1.8)
	Long	2.1 (1.0–3.3)	1.6 (0.8–2.4)	1.6 (0.8–2.4)	1.4 (0.7–2.0)
55	Short	1.9 (1.0–2.9)	1.2 (0.6–1.8)	1.7 (1.0–2.4)	1.0 (0.6–1.4)
	Medium	1.4 (0.6–2.2)	0.7 (0.3–1.2)	1.5 (1.0–2.0)	0.8 (0.5–1.1)
	Long	1.1 (0.3–2.0)	0.8 (0.3–1.3)	1.0 (0.4–1.5)	0.7 (0.3–1.1)
60	Short	0.6 (0.0–1.3)	0.4 (0.1–0.8)	0.6 (0.1–1.0)	0.3 (0.1–0.6)
	Medium	0.4 (–0.1–0.8)	0.3 (0.0–0.5)	0.5 (0.2–0.8)	0.2 (0.1–0.4)
	Long	0.3 (–0.2–0.8)	0.2 (0.0–0.5)	0.3 (0.0–0.6)	0.2 (0.0–0.4)

a Educational duration: Short: early childhood education, primary education, or Shorter secondary education; Medium: upper secondary education, postsecondary, nontertiary education, or short-cycle tertiary education; Long: bachelor’s degree or equivalent, master’s degree or equivalent or doctoral degree or equivalent.

**Table 3b:** Working years lost (WYL) in years and 95% confidence intervals (CI) of **participants living alone** with type 1 (T1) and type 2 (T2) diabetes compared to those without by sex, age and education.

Age	Education a	Women		Men	
	
		T1 compared to controls	T2 compared to controls	T1 compared to controls	T2 compared to controls
			
		WYL (95% CI)	WYL (95% CI)	WYL (95% CI)	WYL (95% CI)
35	Short	6.3 (3.4–9.2)	6.3 (3.9–8.7)	5.3 (2.9–7.7)	6.5 (4.2–8.9)
	Medium	6.6 (4.5–8.8)	4.1 (2.4–5.8)	6.1 (4.6–7.5)	6.7 (5.0–8.5)
	Long	5.1 (2.7–7.6)	2.4 (0.6–4.3)	4.9 (2.9–6.9)	6.5 (4.1–8.9)
40	Short	5.7 (3.3–8.1)	5.6 (3.6–7.5)	4.8 (3.0–6.6)	4.4 (2.8–6.0)
	Medium	5.4 (3.7–7.2)	3.6 (2.2–5.0)	5.5 (4.2–6.7)	4.2 (3.0–5.4)
	Long	4.4 (2.4–6.4)	2.5 (0.9–4.1)	4.2 (2.6–5.9)	4.4 (2.7–6.1)
45	Short	4.4 (2.5–6.3)	4.4 (2.9–5.9)	4.2 (2.8–5.6)	2.6 (1.4–3.7)
	Medium	4.3 (2.8–5.7)	2.7 (1.6–3.8)	4.4 (3.4–5.4)	2.8 (2.0–3.6)
	Long	3.6 (1.9–5.3)	2.2 (0.9–3.5)	3.6 (2.2–5.0)	3.3 (2.0–4.6)
50	Short	3.1 (1.6–4.5)	2.8 (1.8–3.9)	3.2 (2.1–4.3)	1.6 (0.8–2.4)
	Medium	3.1 (1.9–4.2)	1.8 (1.0–2.6)	3.3 (2.5–4.0)	1.7 (1.1–2.2)
	Long	2.6 (1.2–3.9)	1.6 (0.6–2.6)	2.7 (1.6–3.8)	2.2 (1.3–3.1)
55	Short	1.9 (0.8–3.0)	1.6 (0.9–2.3)	1.9 (1.1–2.7)	1.0 (0.5–1.5)
	Medium	1.8 (0.9–2.8)	1.0 (0.4–1.5)	2.0 (1.4–2.6)	0.9 (0.5–1.3)
	Long	1.4 (0.4–2.4)	0.9 (0.2–1.5)	1.6 (0.8–2.4)	1.1 (0.5–1.7)
60	Short	0.7 (–0.1–1.2)	0.6 (0.2–1.0)	0.7 (0.1–1.2)	0.4 (0.1–0.7)
	Medium	0.5 (–0.1–1.1)	0.4 (0.1–0.8)	0.7 (0.3–1.1)	0.3 (0.1–0.5)
	Long	0.4 (–0.2–1.1)	0.3 (–0.2–0.8)	0.5 (0.1–1.0)	0.3 (0.0–0.5)

a Educational duration: Short: early childhood education, primary education, or Shorter secondary education; Medium: upper secondary education, postsecondary, nontertiary education, or short-cycle tertiary education; Long: bachelor’s degree or equivalent, master’s degree or equivalent or doctoral degree or equivalent.

### Number of years in specific labor market transitions

Online supplementary tables S1–5 (www.sjweh.fi/article/3972) show the number of years people with T1 and T2 diabetes spend in the specific transitions compared with matched controls without diabetes.

*Sickness absence*. At age 35–55 years, both cohabitant men and men living alone with T1 diabetes and medium education spend significantly more time in sickness absence compared to people without diabetes (table S1). No significant differences were found in the number of years in sickness absence among people with diabetes compared to controls aged 60–65 years. We found no significant differences between the number of years in sickness absences between T1 and T2 diabetes.

*Unemployment*. With some exceptions, women with T2 diabetes, spend significantly more time in unemployment throughout the work lifespan compared to those without diabetes. These results were most consistent among women, who were living alone or had short or medium periods of education (table S2). Cohabitant men with T2 diabetes and medium education also spend significantly more time in unemployment from age 35– 55 years. No significant differences were found between the diabetes types.

*Disability pension*. With a few exceptions, at age 35–55 years, people with diabetes spend significantly more time in disability pension compared to people without diabetes (table S3). Men and women who were living alone and with short or medium years of education had the highest number of years with disability pension. At most age intervals, people with T1 diabetes spend significantly more years in disability pension, than people with T2 diabetes. These significant differences were most consistent among people with short or medium years of education.

*Temporary absence*. No significant differences were found in the WYL for people with diabetes compared to those without diabetes or between diabetes types (table S4).

*Death*. Regardless of cohabitation status, men with T2 diabetes had significantly more WYL because of death in the age range 35–55 compared to people without diabetes (table S5). With one exception, men who were living alone with T1 diabetes and women with T1 or T2 diabetes living alone, primarily with short education years, had more years lost to death.

### Number of people not registered in DREAM with a yearly income of less than 50 000 DK

Of 1 486 541 individuals without any registered social benefits in DREAM in 2016, 1.9% (28 307) had a yearly income of 0–50 000 DKK (data not included). These analyses excluded people who emigrated or died or were outside the age range 18–64 years in 2016.

## Discussion

The work life losses among people with T1 and T2 diabetes over a 30-year work lifespan are substantial and characterized by social disparities. Social disparities were observed in WLE among both people with and without diabetes. However, in comparison to people without diabetes the educational disparities in WLE were larger for people with diabetes throughout the work lifespan. People with diabetes with lower education had the shortest WLE and most WYL throughout their work life compared to people without diabetes. For example, at age 35, cohabitant women with T1 diabetes and lower education lost up to 8 years; the equivalent for men was 7 years. The WYL were 4 years among women and 3 years among men with higher education.

Previous studies applying a similar WLE methodology have demonstrated that 55-year-old workers with poor health have an average WYL of up to 1.4 years (18), whereas WYL is 1.1 years for depressive symptoms (15, 16). In comparison, our study showed similar results for people with diabetes. However, the theoretical model applied in this study combined with the most recent WLE methodology showed that social factors such as educational and cohabitant status are important to consider along with health factors. Our study showed that people with diabetes spend significantly more time in sickness absence, unemployment, disability pension and death, but not temporary absence (eg, maternity leave, student). The significant differences in number of years spent in these states for people with diabetes compared to controls varied according to sex, types of diabetes, cohabitant and educational status. For example, at age 35–55 men with T1 diabetes with medium education, spend significantly more time in sickness absence than people without diabetes. Women with T2 diabetes – particularly if they live alone – spend more time in unemployment compared to people without diabetes. Overall, people with diabetes spend more years in disability pension, compared to those without diabetes.

Although this is the first study to examine WLE and WYL among people with diabetes, our results suggest that WLE and WYL was highly influenced by social disparities. In keeping with a previous study examining the labor market consequences of diabetes (12), we found no difference in the number of years lost between people with T1 and T2 diabetes of similar age. However, people with T1 diabetes spend significantly more years in disability pension than those with T2 diabetes. Gender differences in WLE and WYL appeared most prominent among cohabitating men and women, which may reflect a general trend observed in high-income countries that cohabitating women are younger than their spouses and more motivated to retire earlier if their spouse retires (46).

The impact of educational status on work-related outcomes has been demonstrated among people with T2 diabetes (24, 31), but less is known on the topic in relation to T1 diabetes. Our results suggest that educational status affects health- and work-related outcomes of people with both T1 and T2 diabetes. From a life-course perspective, our results also suggest that longer periods of education can be protective over the entire work lifespan. We used education as a measure of social inequalities. The impact of short education years on WLE may reflect the poor physical and psychosocial working environment of people with less education as demonstrated in recent studies of WLE (19) and sickness absence (47). Although the impact of social inequalities on WLE should be understood in relation to the other demographic, social and health factors (eg, age, gender, cohabitant status, diabetes types), our results may nevertheless suggest that poor working environment associated with jobs requiring lower or no education may be important intervention targets.

Our study has important limitations. We were not able to identify individuals with T2 diabetes who did not take medication or had not received hospital in- or out-patient care. Consequently, the WLE cannot be generalized to these cases. Although we were able to predict WLE from the entire Danish population, our predictions were based on retrospective data without taking into consideration potential improvements in diabetes outcomes among future generations. The estimates were also based on the highest achieved educational level at age 35 and did not account for changes in educational status after age 35.

The definition of work as time without receipt of social transfer payments may overestimate the results for WLE because this time may also include individuals relying on other economic means, eg, savings. Although the high living expenses in Denmark, combined with high taxes on income and capital, makes self-financed retirement or unemployment before the age of 60 rare, the risk of misclassification cannot be ruled out. However, our analysis suggests that the risk of misclassifying is <2%. Also, we were only able to account for long-term sickness absence and not for shorter spells of sickness absence, which may underestimate the overall impact of diabetes on WLE.

When controlling for differences in comorbidities, we excluded conditions that had high prevalence in diabetes populations since we considered them mental or physical consequences of living with diabetes (eg. kidney disease, retinopathy, depression) and not confounders. Including these conditions in the inverse probability weights would be likely to decrease estimates of the impact of diabetes.

The predictive nature of the WLE estimates relies on the assumption of proportionality underlying Cox regression modeling and that future labor market affiliation can be predicted by the current labor market transitions. Those assumptions are valid only if the circumstances, such as economic conditions, of the follow-up period are comparable to both the study period reported here and the Danish system.

The number of years lost throughout the work life­span for individuals with T1 or T2 diabetes is substantial compared with people without diabetes and also characterized by larger social disparities than people without diabetes. The results highlight the need for new preventive strategies to prevent and manage diabetes-associated disability. Although work is an important aspect of quality of life and an important setting for diabetes management, no occupational health guidelines exists to prevent and manage diabetes-associated work disability. In particular, the results highlight the need to target individuals with short periods of education to alleviate the individual and societal consequences of living with T1 and T2 diabetes. The new approach to examining WYL applied here enables further identification of prevention targets and strategies taking into account specific gender, types of diabetes, educational and cohabitation profiles at different ages throughout the work lifespan.

## Supplementary material

Supplementary material
