# Supplementary material for: Working life expectancies among individuals with type 1 and type 2 diabetes over a 30-year period
Source: Scand J Work Environ Health. 2021 Sep 30;47(7):540–9. doi: 10.5271/sjweh.3972 (PMC8504164; doi:10.5271/sjweh.3972)
Supplement: Supplementary material [file SJWEH-47-540-S001.pdf]

Working life expectancies among individuals with type 1 and type 2 diabetes over a 30-year period [1]

by Mette A Nexø, PhD,[2]Jacob Pedersen, PhD, Bryan Cleal, PhD, Ingelise Andersen, PhD, Jakob B Bjørner, PhD

1. *Supplementary material*
2. *Correspondence to: Mette A. Nexø, Steno Diabetes Center Copenhagen, Department of Education, Gentofte, Denmark [E-mail, mette.andersen.nexoe@regionh.dk]*

Supplementary table S1. Number of years in *Long Term Sickness Absence (LTSA)* with 95% confidence intervals of people with diabetes, compared to people without diabetes, by cohabitant status, sex, age, and education

Supplementary table S2. Number of years in *unemployment (Uemployed)* with 95% confidence intervals of people with diabetes, compared to people without diabetes, by cohabitant status, sex, age, and education

Supplementary table S3. Number of years in *disability pension (DisPen)* in years with 95% confidence intervals of people with diabetes compared to people without diabetes, by cohabitant status, sex, age, and education

Supplementary table S4. Number of years in *temporary absence (temporary)* with 95% confidence intervals of people with diabetes, compared to people without diabetes by cohabitant status, sex, age, and education

Supplementary table S5. Number of years due to *death* with 95% confidence intervals in people with diabetes compared with people without diabetes by cohabitant status, sex, age, and education

Supplementary figure S1. Working life expectancies in cohabitant men and women with diabetes and controls by three educational durations (long, medium, short)

Supplementary table S1. Number of years in *Long Term Sickness Absence (LTSA)* with 95% confidence intervals of people with diabetes, compared to people without diabetes, by cohabitant status, sex, age, and education

		Cohabitant – Women: Type 1 diabetes	Cohabitant – Women: Type 2 diabetes	Cohabitant – Men: Type 1 diabetes	Cohabitant – Men: Type 2 diabetes	Living alone – Women: Type 1 diabetes	Living alone – Women: Type 2 diabetes	Living alone – Men: Type 1 diabetes	Living alone – Men: Type 2 diabetes
Age	Edu.	LTSA (95% CI)	LTSA (95% CI)	LTSA (95% CI)	LTSA (95% CI)	LTSA (95% CI)	LTSA (95% CI)	LTSA (95% CI)	LTSA (95% CI)
35	S	0.5 (-0.9-1.9)	0.3 (-0.8-1.4)	0.9 (-0.2-2.1)	0.4 (-0.5-1.2)	0.4 (-1.0-1.8)	0.0 (-1.0-1.0)	0.6 (-0.4-1.5)	0.0 (-0.7-0.7)
	M	0.8 (-0.2-1.8)	0.7 (-0.1-1.5)	**0.9 (0.2-1.5)**	0.3 (-0.2-0.8)	0.7 (-0.4-1.8)	0.6 (-0.3-1.4)	**0.7 (0.1-1.4)**	0.1 (-0.4-0.6)
	L	0.7 (-0.5-1.8)	0.7 (-0.2-1.6)	0.5 (-0.1-1.2)	0.4 (-0.2-1.0)	0.7 (-0.6-1.9)	0.8 (-0.2-1.9)	0.4 (-0.3-1.1)	0.2 (-0.4-0.8)
40	S	0.5 (-0.6-1.7)	0.3 (-0.6-1.1)	0.9 (0.0-1.8)	0.3 (-0.3-1.0)	0.4 (-0.7-1.6)	0.0 (-0.8-0.8)	0.6 (-0.2-1.3)	0.1 (-0.4-0.6)
	M	0.7 (-0.2-1.6)	0.6 (0.0-1.2)	**0.8 (0.2-1.4)**	0.3 (0.0-0.7)	0.7 (-0.3-1.6)	0.5 (-0.2-1.2)	**0.7 (0.1-1.2)**	0.2 (-0.2-0.6)
	L	0.6 (-0.4-1.6)	0.6 (-0.1-1.4)	0.5 (-0.1-1.1)	0.3 (-0.2-0.8)	0.6 (-0.5-1.6)	0.7 (-0.2-1.6)	0.4 (-0.2-1.1)	0.2 (-0.3-0.7)
45	S	0.5 (-0.4-1.4)	0.3 (-0.4-1.0)	0.8 (0.0-1.5)	0.4 (-0.1-0.8)	0.4 (-0.5-1.3)	0.1 (-0.5-0.7)	0.5 (-0.2-1.1)	0.2 (-0.2-0.7)
	M	0.6 (-0.1-1.4)	0.5 (0.0-1.0)	**0.7 (0.2-1.2)**	0.3 (0.0-0.6)	0.6 (-0.2-1.4)	0.4 (-0.1-1.0)	**0.7 (0.2-1.1)**	0.2 (-0.1-0.5)
	L	0.6 (-0.3-1.4)	0.5 (-0.1-1.2)	0.5 (0.0-1.0)	0.3 (-0.1-0.7)	0.5 (-0.4-1.4)	0.6 (-0.1-1.4)	0.4 (-0.2-1.0)	0.2 (-0.2-0.6)
50	S	0.4 (-0.3-1.2)	0.2 (-0.2-0.7)	0.6 (0.0-1.2)	0.3 (0.0-0.6)	0.4 (-0.3-1.1)	0.1 (-0.3-0.5)	0.4 (-0.1-1.0)	0.2 (-0.1-0.6)
	M	0.5 (-0.1-1.1)	0.4 (0.0-0.7)	**0.6 (0.2-1.0)**	0.3 (0.0-0.5)	0.5 (-0.2-1.2)	0.3 (-0.1-0.7)	**0.6 (0.2-1.0)**	0.2 (-0.2-0.4)
	L	0.4 (-0.2-1.1)	0.4 (-0.1-0.9)	0.4 (0.0-0.8)	0.2 (-0.1-0.5)	0.4 (-0.3-1.1)	0.5 (-0.1-1.1)	0.4 (-0.1-0.8)	0.2 (-0.1-0.5)
55	S	0.3 (-0.2-0.8)	0.1 (-0.5-0.7)	0.5 (0.0-0.9)	0.2 (0.0-0.5)	0.3 (-0.3-0.8)	0.1 (-0.2-0.4)	0.4 (-0.1-0.8)	0.2 (-0.1-0.4)
	M	0.3 (-0.1-0.8)	0.2 (0.0-0.5)	**0.4 (0.1-0.7)**	0.2 (0.0-0.3)	0.4 (-0.2-0.9)	0.2 (-0.1-0.5)	**0.4 (0.1-0.7)**	0.1 (0.0-0.3)
	L	0.3 (-0.2-0.8)	0.2 (-0.1-0.6)	0.3 (0.0-0.6)	0.1 (-0.1-0.3)	0.3 (-0.3-0.9)	0.3 (-0.1-0.7)	0.3 (0.0-0.7)	0.1 (-0.1-0.3)
60	S	0.1 (-0.2-0.4)	0.0 (-0.1-0.2)	0.2 (0.0-0.5)	0.1 (0.0-0.2)	0.1 (-0.2-0.4)	0.0 (-0.1-0.2)	0.2 (-0.1-0.5)	0.1 (-0.1-0.2)
	M	0.1 (-0.2-0.4)	0.1 (0.0-0.3)	0.2 (0.0-0.4)	0.1 (0.0-0.2)	0.2 (-0.2-0.5)	0.1 (-0.1-0.3)	0.2 (0.0-0.5)	0.1 (0.0-0.2)
	L	0.1 (-0.2-0.4)	0.1 (-0.1-0.3)	0.1 (0.0-0.3)	0.0 (-0.1-0.1)	0.1 (-0.2-0.5)	0.1 (-0.1-0.3)	0.2 (-0.1-0.4)	0.0 (0.0-0.2)

Edu.: Educational duration. S: Short: early childhood education, primary education, or Shorter secondary education; M: Medium: upper secondary education, post-secondary non-tertiary education, or short-cycle tertiary education; L: Long: bachelor’s degree or equivalent, master’s degree or equivalent or doctoral degree or equivalent.

Note: Significantly differences $p<0.05$ between people with and without diabetes marked with **bold**; Significantly differences between type 1 and type 2 marked with*

Supplementary table S2. Number of years in *unemployment (Uemployed)* with 95% confidence intervals of people with diabetes, compared to people without diabetes, by cohabitant status, sex, age, and education

		Cohabitant				Living alone			
		Women		Men		Women		Men	
		Type 1 diabetes	Type 2 diabetes	Type 1 diabetes	Type 2 diabetes	Type 1 diabetes	Type 2 diabetes	Type 1 diabetes	Type 2 diabetes
Age	Edu.	Unemployed (95% CI)	Unemployed (95% CI)	Unemployed (95% CI)	Unemployed (95% CI)	Unemployed (95% CI)	Unemployed (95% CI)	Unemployed (95% CI)	Unemployed (95% CI)
35	S	1.0 (-1.0-.03)	1.5 (-0.1-3.2)	0.6 (-0.8-2.0)	0.8 (-0.5-2.0)	0.6 (-1.9-3.2)	2.2 (0.0-4.4)	0.3 (-2.2-1.5)	0.3 (-2.0-1.4)
	M	0.7 (-0.4-1.8)	0.8 (-0.1-1.7)	0.6 (-0.1-1.3)	**0.8 (0.1-1.5)**	1.1 (-0.5-2.7)	**1.6 (0.2-2.9)**	0.2 (-0.9-1.2)	0.4 (-0.7-1.4)
	L	0.5 (-0.6-1.6)	0.8 (-0.1-1.7)	0.6 (-0.2-1.4)	0.7 (-0.2-1.5)	0.5 (-1.0-2.0)	1.2 (-0.2-2.5)	0.6 (-0.8-2.0)	0.5 (-0.9-1.9)
40	S	1.0 (-0.6-2.5)	**1.6 (0.2-2.9)**	0.5 (-0.5-1.6)	0.8 (-0.1-1.6)	0.7 (-1.3-2.7)	**2.3 (0.5-4.0)**	0.3 (-1.7-1.2)	0.2 (-1.1-1.4)
	M	0.6 (-0.4-1.5)	0.8 (0.0-1.5)	0.5 (-0.1-1.1)	**0.6 (0.1-1.1)**	1.0 (-0.3-2.3)	**1.5 (0.4-2.5)**	0.2 (-0.7-1.0)	0.4 (-0.4-1.1)
	L	0.5 (-0.4-1.3)	0.8 (0.0-1.6)	0.5 (-0.2-1.2)	0.6 (-0.1-1.2)	0.4 (-0.8-1.7)	1.1 (0.0-2.3)	0.5 (-0.6-1.7)	0.6 (-0.5-1.6)
45	S	0.8 (-0.4-2.0)	**1.2 (0.3-2.2)**	0.4 (-0.4-1.2)	0.6 (-0.1-2.0)	0.7 (-0.9-2.2)	**1.9 (0.6-3.2)**	0.2 (-1.3-0.9)	0.4 (-0.5-1.3)
	M	0.5 (-0.3-1.2)	**0.6 (0.1-1.2)**	0.5 (0.0-1.0)	**0.5 (0.2-0.9)**	0.8 (-0.3-1.9)	**1.2 (0.3-2.1)**	0.2 (-0.5-1.0)	0.4 (-0.2-1.0)
	L	0.4 (-0.4-1.2)	**0.7 (0.1-1.4)**	0.4 (-0.2-1.0)	0.5 (0.0-1.0)	0.4 (-0.7-1.5)	**1.0 (0.1-2.0)**	0.4 (-0.5-1.4)	0.6 (-0.3-1.4)
50	S	0.7 (-0.3-1.6)	**0.9 (0.2-1.5)**	0.4 (-0.3-1.0)	0.4 (0.0-0.8)	0.6 (-0.6-1.9)	**1.5 (0.5-2.5)**	0.1 (-0.9-0.8)	0.3 (-0.3-1.0)
	M	0.3 (-0.3-0.9)	0.4 (0.0-0.9)	0.4 (0.0-0.8)	**0.4 (0.1-0.6)**	0.6 (-0.3-1.5)	**0.9 (0.2-1.5)**	0.2 (-0.4-0.8)	0.2 (-0.2-0.7)
	L	0.3 (-0.3-0.9)	**0.6 (0.1-1.1)**	0.3 (-0.2-0.8)	0.4 (0.0-0.7)	0.3 (-0.6-1.2)	**0.8 (0.1-1.5)**	0.3 (-0.5-1.1)	0.4 (-0.2-1.0)
55	S	0.4 (-0.3-1.1)	-0.2 (-0.9-0.6)	0.2 (-0.2-0.7)	0.3 (0.0-0.5)	0.3 (-0.6-1.3)	**1.0 (0.3-1.6)**	0.2 (-0.7-0.7)	0.2 (-0.3-0.7)
	M	0.2 (-0.3-0.7)	0.3 (0.0-0.6)	0.3 (0.0-0.6)	0**.3 (0.1-0.4)**	0.4 (-0.3-1.1)	0.5 (0.0-1.0)	0.2 (-0.3-0.7)	0.2 (-0.1-0.5)
	L	0.2 (-0.3-0.7)	0.3 (0.0-0.7)	0.2 (-0.1-0.6)	0.2 (0.0-0.5)	0.2 (-0.5-0.9)	0.5 (0.0-1.0)	0.2 (-0.4-0.8)	0.2 (-0.2-0.6)
60	S	0.2 (-0.3-0.6)	0.3 (0.0-0.5)	0.1 (-0.2-0.3)	0.1 (0.0-0.3)	0.2 (-0.4-0.8)	**0.5 (0.1-0.8)**	0.0 (-0.4-0.4)	0.1 (-0.1-0.4)
	M	0.1 (-0.2-0.3)	0.1 (-0.1-0.3)	0.1 (-0.1-0.3)	0.1 (0.0-0.2)	0.1 (-0.3-0.5)	**0.2 (0.0-0.5)**	0.1 (-0.2-0.4)	0.1 (-0.1-0.3)
	L	0.1 (-0.2-0.4)	0.1 (-0.1-0.3)	0.1 (-0.1-0.3)	0.1 (0.0-0.2)	0.1 (-0.3-0.5)	0.1 (-0.1-0.4)	0.1 (-0.2-0.5)	0.1 (-0.1-0.3)

Edu.: Educational duration. S: Short: early childhood education, primary education, or Shorter secondary education; M: Medium: upper secondary education, post-secondary non-tertiary education, or short-cycle tertiary education; L: Long: bachelor's degree or equivalent, master's degree or equivalent or doctoral degree or equivalent.
Note: Significantly differences $p<0.05$ between people with and without diabetes marked with **bold**; Significantly differences between type 1 and type 2 marked with*

Supplementary table S3. Number of years in *disability pension (DisPen)* in years with 95% confidence intervals of people with diabetes compared to people without diabetes, by cohabitant status, sex, age, and education

		Cohabitant						Living alone					
		Women			Men			Women			Men		
		Type 1 diabetes	Type 2 diabetes		Type 1 diabetes	Type 2 diabetes		Type 1 diabetes	Type 2 diabetes		Type 1 diabetes	Type 2 diabetes	
Age	Edu.	DisPen (95% CI)	DisPen (95% CI)		DisPen (95% CI)	DisPen (95% CI)		DisPen (95% CI)	DisPen (95% CI)		DisPen (95% CI)	DisPen (95% CI)	
35	S	**4.7 (2.0-7.4)**	**3.4 (1.3-5.5)**		**3.7 (1.8-5.6)**	**3.8 (1.5-6.1)**		2.8 (0.0-5.7)	**2.8 (0.4-5.3)**		**3.6 (1.1-6.1)**	**4.4 (1.5-7.3)**	
	M	**3.3 (1.9-4.7)**	**1.5 (0.6-2.5)**	*	**2.5 (1.7-3.4)**	**2.3 (1.2-3.5)**		**4.0 (2.4-5.6)**	**1.6 (0.5-2.7)**	*	**4.3 (3.1-5.5)**	**4.4 (2.6-6.1)**	
	L	**2.6 (1.2-4.1)**	**1.0 (0.1-1.9)**		**1.1 (0.4-1.9)**	**1.2 (0.2-2.1)**		**3.1 (1.4-4.7)**	0.3 (0.7-1.3)		**2.7 (1.5-3.9)**	**3.1 (1.2-5.1)**	
40	S	**3.8 (1.8-5.8)**	**2.4 (0.9-4.0)**		**3.0 (1.6-4.4)**	**2.1 (1.0-3.3)**		**2.5 (0.4-4.7)**	**2.2 (0.3-4.0)**		**3.5 (1.7-5.3)**	**2.7 (1.0-4.5)**	
	M	**2.5 (1.4-3.6)**	**1.4 (0.6-2.2)**		**2.2 (1.5-3.0)**	**1.2 (0.5-1.9)**		**3.0 (1.8-4.3)**	**1.4 (0.5-2.4)**	*	**3.9 (2.9-5.0)**	**2.3 (1.2-3.4)**	*
	L	**2.2 (1.0-3.4)**	**0.9 (0.1-1.7)**		**1.0 (0.4-1.7)**	**0.9 (0.2-1.7)**		**2.6 (1.2-4.0)**	0.4 (0.5-1.3)		**2.5 (1.4-3.6)**	**2.3 (1.0-3.6)**	
45	S	**2.5 (1.1-3.9)**	**1.8 (0.7-2.9)**		**2.3 (1.4-3.3)**	**1.1 (0.4-1.8)**	*	**1.7 (0.2-3.2)**	**1.6 (0.3-2.9)**		**2.9 (1.7-4.2)**	**1.3 (0.2-2.3)**	
	M	**1.8 (1.0-2.6)**	**0.9 (0.3-1.5)**		**1.6 (1.1-2.2)**	**0.8 (0.4-1.1)**	*	**2.2 (1.3-3.2)**	**0.9 (0.2-1.6)**	*	**2.9 (2.1-3.7)**	**1.5 (0.9-2.1)**	*
	L	**1.7 (0.8-2.7)**	**0.7 (0.1-1.4)**		**0.9 (0.2-1.5)**	**0.6 (0.1-1.1)**		**2.0 (0.9-3.2)**	0.3 (0.4-1.1)		**2.2 (1.2-3.2)**	**1.6 (0.7-2.5)**	
50	S	**1.5 (0.6-2.5)**	**0.8 (0.2-1.5)**		**1.6 (0.9-2.3)**	**0.6 (0.2-1.0)**	*	**1.1 (0.1-2.1)**	0.7 (0.0-1.4)	*	**2.2 (1.3-3.1)**	0.7 (0.0-1.3)	
	M	**1.3 (0.6-1.9)**	**0.5 (0.1-0.9)**	*	**1.1 (0.7-1.5)**	**0.4 (0.2-0.7)**	*	**1.6 (0.8-2.3)**	**0.5 (0.1-1.0)**	*	**2.1 (1.5-2.7)**	**0.8 (0.4-1.1)**	*
	L	**1.2 (0.5-1.9)**	0.5 (0.0-1.0)		**0.7 (0.2-1.2)**	**0.4 (0.1-0.7)**		**1.4 (0.6-2.3)**	0.2 (-0.3-0.7)		**1.6 (0.8-2.3)**	**1.0 (0.4-1.6)**	
55	S	**0.8 (0.2-1.4)**	0.3 (0.0-0.6)		**0.7 (0.3-1.1)**	**0.3 (0.1-0.5)**		0.6 (0.0-1.2)	0.3 (-0.1-0.6)		**1.0 (0.5-1.6)**	0.3 (0.0-0.6)	
	M	**0.6 (0.2-1.1)**	0.2 (0.0-0.4)		**0.6 (0.3-0.9)**	**0.2 (0.1-0.3)**	*	**0.8 (0.3-1.3)**	0.2 (-0.1-0.4)		**1.1 (0.7-1.5)**	**0.4 (0.2-0.6)**	*
	L	**0.6 (0.1-1.0)**	0.2 (-0.1-0.4)		**0.4 (0.1-0.6)**	0.2 (0.0-0.4)		**0.7 (0.1-1.2)**	0.0 (0.2-0.3)		**0.9 (0.5-1.3)**	**0.5 (0.2-0.8)**	
60	S	0.2 (-0.1-0.6)	0.1 (-0.1-0.2)		0.1 (-0.1-0.3)	0.1 (0.0-0.2)		**0.2 (0.2-0.6)**	0.1 (-0.1-0.2)		0.2 (-0.1-0.5)	0.1 (-0.1-0.2)	
	M	0.1 (-0.1-0.3)	0.1 (0.0-0.2)		0.1 (-0.1-0.2)	0.0 (0.0-0.1)		0.1 (-0.1-0.4)	0.1 (0.0-0.2)		0.2 (0.0-0.4)	0.1 (0.0-0.2)	
	L	0.1 (-0.1-0.4)	0.0(-0.1-0.1)		0.1 (-0.5-0.2)	0.0 (-0.1-0.1)		0.2 (-0.1-0.4)	0.0 (-0.1-0.1)		0.2 (0.0-0.5)	0.1 (-0.1-0.2)	

Edu.: Educational duration. S: Short: early childhood education, primary education, or Shorter secondary education; M: Medium: upper secondary education, post-secondary non-tertiary education, or short-cycle tertiary education; L: Long: bachelor's degree or equivalent, master's degree or equivalent or doctoral degree or equivalent.
Note: Significantly differences $p<0.05$ between people with and without diabetes marked with **bold**; Significantly differences between type 1 and type 2 marked with*

Supplementary table S4. Number of years in *temporary absence (temporary)* with 95% confidence intervals of people with diabetes, compared to people without diabetes by cohabitant status, sex, age, and education

		Cohabitant				Living alone			
		Women		Men		Women		Men	
		Type 1 diabetes	Type 2 diabetes	Type 1 diabetes	Type 2 diabetes	Type 1 diabetes	Type 2 diabetes	Type 1 diabetes	Type 2 diabetes
Age	Edu.	Temporary (95% CI)	Temporary (95% CI)	Temporary (95% CI)	Temporary (95% CI)	Temporary (95% CI)	Temperary (95% CI)	Temporary (95% CI)	Temporary (95% CI)
35	S	-0.4 (-1.1-0.4)	0.0 (-0.8-0.8)	-0.1 (-0.7-0.5)	0.0 (-0.6-0.7)	-0.4 (-1.3-0.4)	0.0 (-0.9-0.9)	-0.4 (-1.0-0.2)	-0.1 (-0.9-0.7)
	M	-0.1 (-0.7-0.5)	0.0 (-0.5-0.5)	-0.1 (-0.5-0.2)	0.1 (-0.4-0.5)	-0.4 (-1.0-0.3)	-0.2 (-0.7-0.4)	-0.5 (-1.0-0.0)	-0.3 (-0.9-0.4)
	L	-0.3 (-1.0-0.4)	0.0 (-0.6-0.6)	-0.3 (-0.8-0.3)	-0.2 (-0.8-0.5)	-0.5 (-1.1-0.2)	-0.4 (0.9-0.2)	-1.1 (-2.0--0.3)	-0.7 (-1.9-0.4)
40	S	-0.2 (-0.7-0.4)	0.0 (-0.6-0.5)	-0.1 (-0.5-0.4)	0.0 (-0.4-0.3)	-0.2 (-0.8-0.4)	-0.1 (-0.7-0.6)	-0.3 (-0.7-0.2)	-0.1 (-0.6-0.4)
	M	-0.1 (-0.5-0.3)	-0.1 (-0.4-0.2)	-0.1 (-0.4-0.2)	0.0 (-0.3-0.3)	-0.2 (-0.7-0.3)	-0.2 (-0.6-0.2)	-0.4 (-0.8-0.1)	-0.1 (-0.6-0.3)
	L	-0.2 (-0.6-0.3)	-0.1 (-0.5-0.3)	0.2 (-0.7-0.2)	-0.1 (-0.6-0.4)	-0.3 (-0.7-0.2)	-0.2 (-0.6-0.1)	-0.8 (-1.5--0.1)	-0.3 (-1.3-0.6)
45	S	-0.1 (-0.5-0.4)	0.0 (-0.4-0.4)	-0.1 (-0.4-0.3)	0.0 (-0.3-0.2)	-0.1 (-0.6-0.5)	0.0 (-0.4-0.5)	-0.2 (-0.5-0.1)	-0.1 (-0.4-0.2)
	M	0.0 (-0.4-0.3)	-0.1 (-0.3-0.2)	0.0 (-0.3-0.2)	0.0 (-0.2-0.2)	-0.1 (-0.5-0.3)	-0.1 (-0.4-0.1)	-0.2 (-0.5-0.1)	-0.1 (-0.4-0.2)
	L	-0.1 (-0.5-0.3)	-0.1 (-0.4-0.2)	-0.2 (-0.5-0.2)	0.0 (-0.4-0.4)	-0.1 (-0.5-0.3)	-0.2 (-0.4-0.1)	0.5 (-1.1-0.1)	0.0 (-0.8-0.7)
50	S	0.0(-0.3-0.3)	0.0 (-0.3-0.3)	0.0 (-0.3-0.2)	0.0 (-0.2-0.2)	0.0 (-0.4-0.3)	0.0 (-0.3-0.3)	-0.1 (-0.4-0.1)	0.0 (-0.3-0.2)
	M	0.0 (-0.3-0.2)	-0.1 (-0.2-0.1)	0.0 (-0.2-0.2)	0.0 (-0.1-0.2)	-0.1 (-0.3-0.2)	-0.1 (-0.3-0.1)	-0.1 (-0.4-0.2)	0.0 (-0.2-0.2)
	L	-0.1 (-0.3-0.2)	0.0 (-0.3-0.2)	-0.1 (-0.4-0.2)	0.0 (-0.2-0.3)	-0.1 (-0.4-0.2)	-0.1 (-0.3-0.1)	-0.3 (-0.8-0.2)	0.0 (-0.5-0.6)
55	S	0.0 (-0.2-0.3)	0.0 (-0.3-0.4)	0.0 (-0.2-0.3)	0.0 (-0.1-0.2)	0.0 (-0.3-0.4)	0.0 (-0.3-0.3)	0.0 (-0.3-0.3)	0.0 (-0.2-0.2)
	M	0.1 (-0.3-0.4)	0.0 (-0.2-0.1)	0.0 (-0.2-0.2)	0.0(-0.1-0.1)	0.0 (-0.3-0.4)	-0.1 (-0.2-0.1)	0.0 (-0.3-0.3)	0.0 (-0.1-0.2)
	L	0.0 (-0.2-0.1)	0.0 (-0.2-0.1)	-0.1 (-0.3-0.2)	0.0 (-0.2-0.2)	-0.1 (-0.3-0.2)	-0.1 (-0.2-0.1)	-0.1 (-0.5-0.3)	0.0 (-0.3-0.4)
60	S	0.0 (-0.2-0.1)	0.0 (-0.2-0.2)	0.0 (-0.2-0.2)	0.0 (-0.1-0.1)	0.0 (-0.3-0.3)	0.0 (-0.3-0.3)	0.0 (-0.3-0.3)	0.0 (-0.2-0.2)
	M	0.0 (-0.2-0.2)	0.0 (-0.1-0.0)	0.0 (-0.1-0.2)	0.0 (-0.1-0.1)	0.0 (-0.3-0.4)	0.0 (-0.1-0.1)	0.0 (-0.3-0.3)	0.0 (-0.1-0.1)
	L	0.0 (-0.2-0.1)	0.0(-0.1-0.1)	0.0 (-0.2-0.1)	0.0 (-0.1-0.1)	0.0 (-0.4-0.4)	0.0 (-0.1-0.1)	-0.1 (-0.3-0.2)	0.0 (-0.2-0.2)

Edu.: Educational duration. S: Short: early childhood education, primary education, or Shorter secondary education; M: Medium: upper secondary education, post-secondary non-tertiary education, or short-cycle tertiary education; L: Long: bachelor's degree or equivalent, master's degree or equivalent or doctoral degree or equivalent.

Note: Significantly differences $p<0.05$ between people with and without diabetes marked with **bold**; Significantly differences between type 1 and type 2 marked with*

Supplementary table S5. Number of years due to *death* with 95% confidence intervals in people with diabetes compared with people without diabetes by cohabitant status, sex, age, and education

		Cohabitant				Living alone			
		Women		Men		Women		Men	
		Type 1 diabetes	Type 2 diabetes	Type 1 diabetes	Type 2 diabetes	Type 1 diabetes	Type 2 diabetes	Type 1 diabetes	Type 2 diabetes
Age	Edu.	Death (95% CI)	Death (95% CI)	Death (95% CI)	Death (95% CI)	Death (95% CI)	Death (95% CI)	Death (95% CI)	Death (95% CI)
35	S	0.7 (-0.3-1.7)	0.5 (-0.2-1.1)	0.6 (-0.2-1.3)	**1.2 (0.2-2.2)**	**1.3 (0.2-2.4)**	0.5 (0.0-1.1)	0.9 (0.0-1.9)	**1.2 (0.3-2.1)**
	M	0.3 (-0.1-0.8)	0.2 (-0.1-0.5)	0.5 (0.0-0.9)	**1.1 (0.5-1.8)**	0.5 (0.0-1.0)	0.2 (-0.1-0.5)	**0.8 (0.3-1.4)**	**1.2 (0.5-1.9)**
	L	0.3 (-0.2-0.8)	0.1 (-0.2-0.4)	**0.6 (0.1-1.2)**	**1.0 (0.2-1.8)**	0.7 (-0.1-1.5)	0.1 (-0.2-0.4)	**1.2 (0.4-1.9)**	**1.3 (0.3-2.2)**
40	S	0.8 (-0.1-1.7)	0.6 (0.0-1.2)	0.5 (-0.1-1.1)	**1.0 (0.3-1.6)**	**1.3 (0.3-2.3)**	**0.7 (0.1-1.2)**	**0.8 (0.1-1.5)**	**1.1 (0.4-1.8)**
	M	0.3 (-0.1-0.8)	0.2 (-0.1-0.5)	0.4 (0.0-0.7)	**0.9 (0.5-1.4)**	**0.6 (0.1-1.1)**	0.2 (-0.1-0.5)	**0.7 (0.3-1.2)**	**1.2 (0.7-1.7)**
	L	0.2 (-0.2-0.6)	0.1 (-0.2-0.4)	0.5 (0.0-0.9)	**0.8 (0.2-1.3)**	0.5 (-0.1-1.1)	0.1 (-0.2-0.4)	**0.9 (0.3-1.5)**	**1.0 (0.4-1.7)**
45	S	0.7 (0.0-1.4)	0.5 (0.0-1.0)	0.5 (0.0-1.0)	**0.5 (0.1-0.9)**	**1.2 (0.4-2.0)**	**0.6 (0.1-1.1)**	**0.8 (0.3-1.4)**	**0.6 (0.2-1.0)**
	M	0.3 (-0.1-0.6)	0.1 (-0.1-0.3)	0.3 (0.0-0.6)	**0.6 (0.3-0.8)**	0.5 (0.0-0.9)	0.2 (-0.1-0.4)	**0.6 (0.2-1.0)**	**0.7 (0.4-1.1)**
	L	0.2 (-0.2-0.5)	0.1 (-0.1-0.3)	0.4 (0.0-0.8)	**0.5 (0.1-0.8)**	0.4 (-0.1-0.9)	0.1 (-0.2-0.3)	**0.7 (0.2-1.2)**	**0.7 (0.2-1.1)**
50	S	0.4 (0.0-0.9)	0.3 (0.0-0.6)	0.3 (0.0-0.7)	**0.3 (0.1-0.5)**	**0.7 (0.2-1.2)**	**0.4 (0.1-0.7)**	**0.6 (0.1-1.0)**	**0.4 (0.1-0.6)**
	M	0.2 (-0.1-0.4)	0.1 (-0.1-0.3)	0.3 (0.0-0.6)	**0.3 (0.2-0.5)**	0.3 (0.0-0.6)	0.1 (0.0-0.3)	**0.4 (0.1-0.7)**	**0.4 (0.2-0.6)**
	L	0.1 (-0.2-0.4)	0.1 (-0.1-0.3)	0.3 (-0.1-0.6)	**0.3 (0.1-0.6)**	0.3 (-0.1-0.7)	0.1 (-0.2-0.3)	**0.5 (0.1-0.9)**	**0 .5 (0.2-0.8)**
55	S	0.3 (0.0-0.6)	0.2 (0.0-0.3)	0.2 (0.0-0.4)	**0.2 (0.1-0.3)**	**0.5 (0.1-0.9)**	0.2 (0.0-0.4)	**0.4 (0.1-0.7)**	**0.3 (0.1-0.4)**
	M	0.1 (-0.1-0.3)	0.1 (0.0-0.2)	0.1 (0.0-0.2)	**0.1 (0.1-0.2)**	0.2 (0.0-0.4)	0.1 (0.0-0.2)	**0.2 (0.1-0.4)**	**0.2 (0.1-0.3)**
	L	0.0 (-0.1-0.2)	0.1 (-0.1-0.2)	0.1 (-0.1-0.3)	0.1 (0.0-0.3)	0.1 (-0.1-0.4)	0.1 (-0.1-0.2)	0.2 (0.0-0.4)	0.2 (0.0-0.3)
60	S	0.2 (-0.1-0.4)	0.1 (0.0-0.2)	0.1 (0.0-0.2)	0.0 (0.0-0.1)	0.3 (0.0-0.5)	0.1 (0.0-0.2)	**0.2 (0.1-0.4)**	0.1 (0.0-0.1)
	M	0.1 (-0.1-0.2)	0.0 (0.0-0.1)	0.1 (0.0-0.1)	0.1 (0.0-0.1)	0.1 (-0.1-0.3)	0.0 (0.0-0.1)	0.1 (0.0-0.2)	0.0 (0.0-0.1)
	L	0.0 (-0.1-0.1)	0.0 (0.0-0.1)	0.1 (-0.1-0.2)	0.0 (-0.1-0.1)	0.0 (-0.1-0.1)	0.0 (-0.1-0.1)	0.1 (0.0-0.2)	0.0 (0.0-0.1)

Edu.: Educational duration. S: Short: early childhood education, primary education, or Shorter secondary education; M: Medium: upper secondary education, post-secondary non-tertiary education, or short-cycle tertiary education; L: Long: bachelor's degree or equivalent, master's degree or equivalent or doctoral degree or equivalent.

Note: Significantly differences $p<0.05$ between people with and without diabetes marked with **bold**; Significantly differences between type 1 and type 2 marked with*

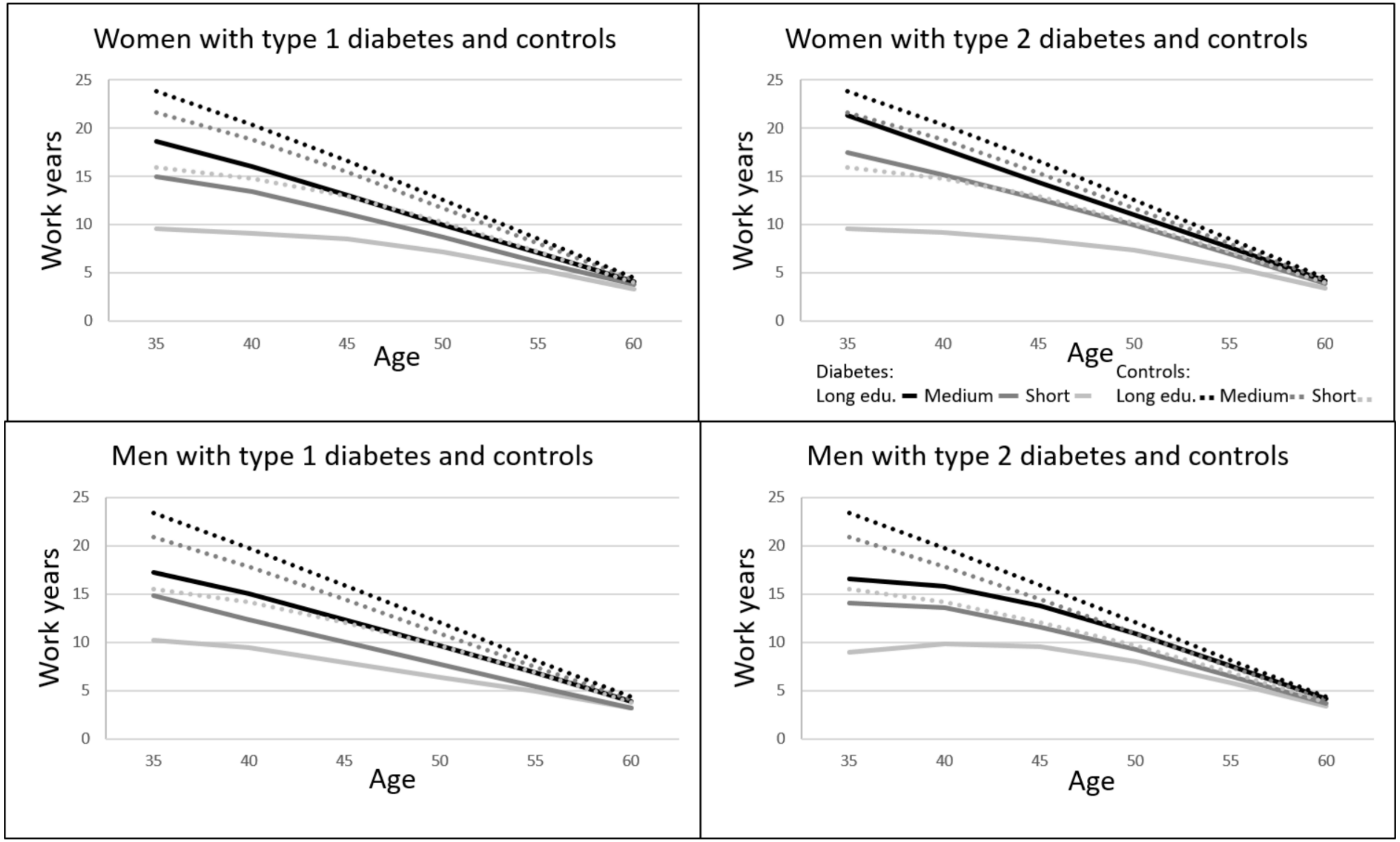

Supplementary figure S1. Working life expectancies for men and women livingalone with diabetes and controls by three educational durations (long, medium, short)
